# Potential of internet‐delivered PCIT for ASD in the COVID‐19 era: A pilot study

**DOI:** 10.1111/ped.14699

**Published:** 2021-09-07

**Authors:** Miyuki Matano, Yukifumi Monden, Koyuru Kurane, Masako Kawasaki, Toshiko Kamo

**Affiliations:** ^1^ Department of Pediatrics Jichi Medical University Tochigi Japan; ^2^ Department of Pediatrics International University of Health and Welfare Tochigi Japan; ^3^ Japan PCIT Training Center Tokyo Japan

**Keywords:** Internet‐delivered parent‐child interaction therapy, telehealth, neurodevelopmental disorders

Autism spectrum disorder (ASD) is neurodevelopmental disorder characterized by core deficits in social interaction and communication. ASD occurs in 0.8–2.0% of school‐aged children, some experiencing comorbid disruptive behaviors.^1^ Parent‐Child Interaction Therapy^2^ (PCIT) is an evidence‐based treatment for disruptive behavior disorders in children aged 2 to 7 years and provides support for caregivers. Multiple clinical studies on using PCIT for children with ASD and their caregivers have reported positive effects.^3^ PCIT aims to strengthen the relationship between the caregiver and child and to improve child compliance. Clinic‐based PCIT reduces child problem behaviors through live coaching of caregiver‐child interactions using a one‐way mirror with a microphone and earphones. In western countries, PCIT is provided in various settings, including homes and hospitals (clinic‐based), but also online, depending on the needs of patients. While clinic‐based PCIT started in Japan about 10 years ago, Internet‐delivered PCIT (I‐PCIT) was implemented in 2020 because of contact limitations created by the COVID‐19 pandemic. To date, there has been only one case report (a maltreatment case) on the efficacy of I‐PCIT in Japan,^4^ therefore an accumulation of data from more studies is needed. This study compared the efficacy of three, clinic‐based PCIT cases and one I‐PCIT case in children with ASD.

This study was approved by the ethics committee of the International University of Health and Welfare. Three parent–child dyads were classified as clinic‐based PCIT and one dyad as I‐PCIT. The four male children for the study from the International University of Health and Welfare (Nasushiobara, Tochigi, Japan), ranged in age from 3 to 5 years (mean 3.6 years, median 3.0 years) and were diagnosed with ASD, based on the DSM‐5 criteria. While clinic‐based PCIT provides live parent coaching in a clinic, I‐PCIT uses a webcam to stream parent‐child interactions in real‐time from their home. PCIT is an evaluation‐oriented treatment. Assessments were conducted before and after treatment using observation and standardized questionnaires, mainly the Eyberg Child Behavior Inventory (ECBI). The ECBI was used to assess the children's problematic behaviors. The scale includes 36 items and comprises an intensity scale and a problem scale. The intensity scale measures the frequency of various behaviors on a 7‐point scale and the problem scale classifies behavior as either problematic or not (yes or no). The Japanese version of the ECBI was standardized by Kamo and the cutoff scores are 124 for the intensity scale and 13 for the problem scale in Japan.^5^ The parents completed the ECBI at each session. Initially, pre‐ and posttreatment ECBI intensity and problem scores were compared using a *t*‐test to verify the effect of the whole PCIT treatment session, which included two phases: child‐directed interaction (CDI) and parent‐directed interaction (PDI) for all four cases (Fig. 1a). Second, we compared the rate of improvement in ECBI intensity‐scores before and after each intervention for clinical‐based PCIT and I‐PCIT (Fig. 1b).

**Fig. 1:**
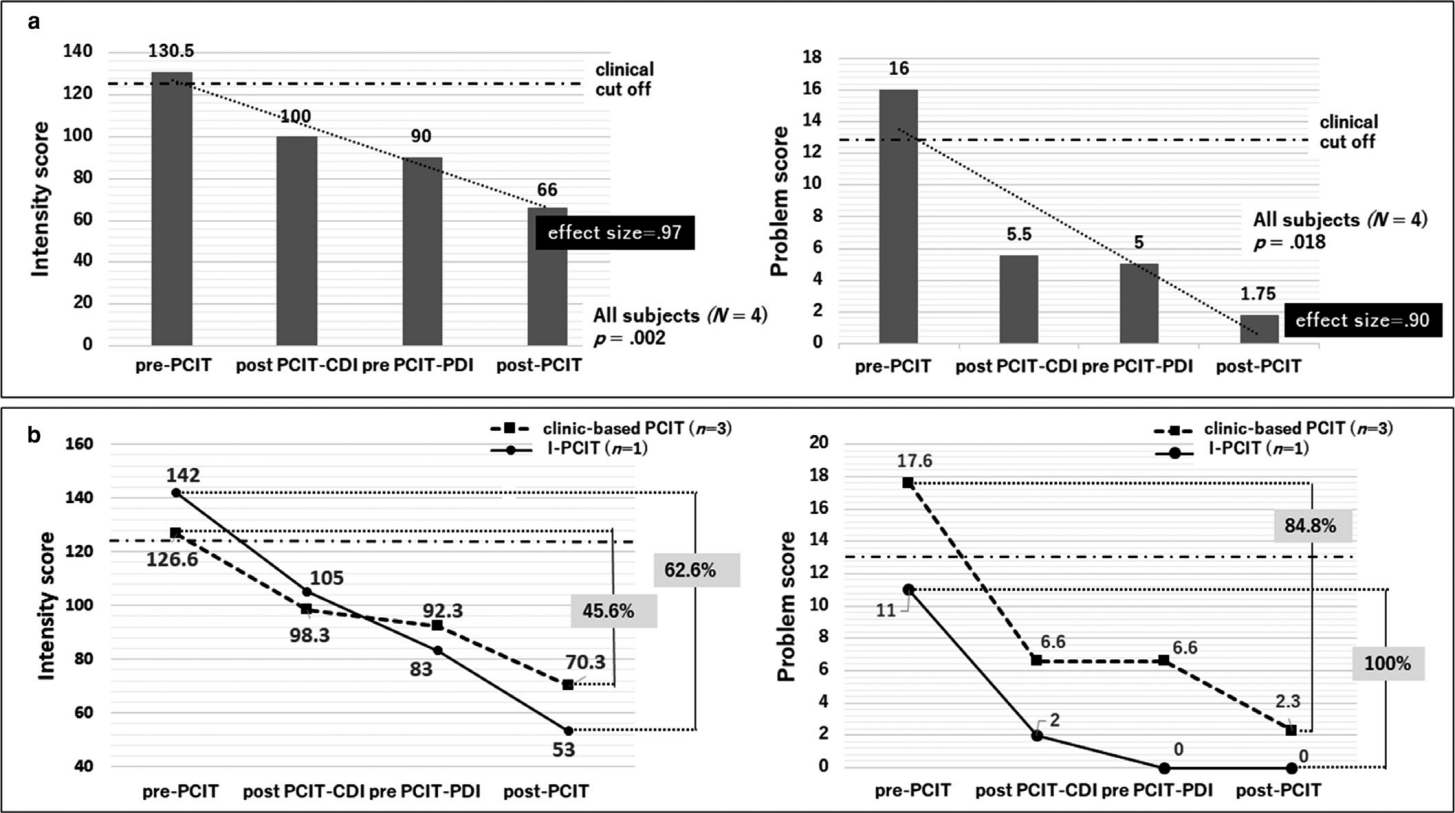
(a) The average of the Eyberg Child Behavior Inventory (ECBI) intensity scores before and after treatment were 130.5 and 66.0. The average of the ECBI problem scores before and after treatment were 16.0 and 1.75. Pre‐ and posttreatment ECBI intensity scores were compared using a *t*‐test to verify the effect of the whole Parent‐Child Interaction Therapy (PCIT) treatment session. There were significant differences before and after treatment for both clinic‐based PCIT and Internet delivered PCIT treatments (I‐PCIT) (*n*= 4, *P *= 0.0002, effect size(*r*) = 0.97; ECBI problem scores: *P *= 0.018, effect size(*r*) = 0.90). (b) Comparison between the rate of improvement in the ECBI intensity and problem scores before and after each intervention for clinical‐based PCIT and I‐PCIT. All dyads showed improvement in ECBI intensity and problem scores, which ranged from 36.8% to 54.8% (mean 45.6, SD 7.3) and 78.5–91.3% (mean 84.8, SD 5.2), respectively, for clinic‐based PCIT (*n* = 3), and 62.6% and 100%, respectively, for I‐PCIT (*n* = 1).

Figure 1a shows the pre‐ and postintervention scores. The average of the ECBI intensity scores before treatment was 130.5 and after treatment was 66.0. The average of ECBI problem scores before treatment was 16.0 and after treatment was 1.7. There were significant differences before and after the entire course of PCIT treatment for both clinic‐based and I‐PCIT treatments (*n *= 4, ECBI intensity scores: *P *= 0.0002, effect size(*r*) = 0.97; ECBI problem scores: *P *= 0.018, effect size(*r*) = 0.90; Fig. 1a). Although the number of subjects was limited, Fig. 1b shows that both clinic‐based PCIT and I‐PCIT exhibited robust effectiveness in statistical validation. All dyads showed improvement in ECBI intensity and problem scores which ranged from 36.8– 54.8% (mean 45.6, SD 7.3) to 78.5–91.3% (mean 84.8, SD 5.2), respectively, for clinic‐based PCIT (*n* = 3) and were 62.6 % and 100%, respectively for I‐PCIT (*n *= 1; Fig. 1b).

PCIT was effective in improving behavioral problems in all cases, regardless of the PCIT type. Using either type of PCIT effectively, seamless PCIT treatment is feasible in the COVID‐19 era. As our previous study,^4^ the current PCIT study may improve the ecological validity of treatment by encouraging the adoption of PCIT at home where therapists can observe the most problematic behaviors. In clinic‐based PCIT, children are sometimes calm and obedient compared to when they are at home, making it difficult for the therapist to intervene. However, for I‐PCIT, advanced preparation, such as Wi‐Fi and video settings in the home, is required.

## Disclosure

The authors declare no conflicts of interest.

## Author contributions

M.M. and Y.M. drafted the initial manuscript and approved the final manuscript. M.K. conceptualized the study and K.K. and T.K. supported the implementation of the PCIT therapy. All authors read and approved the final manuscript.
